# Musical Emotion Modulates Concrete and Abstract Word Processing: Evidence from ERPs

**DOI:** 10.3390/brainsci16080803

**Published:** 2026-07-30

**Authors:** Lili Ming, Limei Wu, Jinqiao Zhang

**Affiliations:** 1 College of Chinese Language and Culture, Jinan University, Guangzhou 510610, China; 2020191203@jsnu.edu.cn (L.M.); iemlw@163.com (L.W.); 2 Key Laboratory of Digital and Intelligent Chinese Language and Culture of Guangdong Province, Guangzhou 510610, China

**Keywords:** emotional priming, concrete words, abstract words, emotional experience, event-related potentials (ERPs)

## Abstract

**Highlights:**

**What are the main findings?**

- Musical emotion modulates the emotional processing of both concrete and abstract words, showing distinct ERP patterns.
- Concrete words are influenced by musical context across multiple processing stages, whereas abstract words show music-related integration mainly at a later stage.

**What are the implications of the main findings?**

- The results support the contextual compensation hypothesis, suggesting that concrete and abstract concepts may benefit differently from external emotional context.
- This study provides ERP evidence for differential mechanisms underlying cross-modal emotional interactions between music and language.

**Abstract:**

**Background/Objectives:** Cross-modal emotional priming effects of music on language have been widely demonstrated, but how musical emotion interacts with different types of conceptual representation remains unclear. This study examined whether emotional musical contexts exert differential influences on the processing of concrete and abstract words, whose processing is thought to depend to different degrees on sensorimotor and affective experience. **Methods:** Two ERP experiments were conducted using a long stimulus-onset asynchrony priming paradigm with two independent groups of participants. In Experiment 1, 24 female participants performed valence judgments on concrete words following positive or negative musical primes. In Experiment 2, 24 female participants completed the same task with abstract words. ERP components associated with early perceptual or attentional processing, semantic processing, and later evaluative or integrative processing were analyzed. **Results:** Musical emotion modulated the processing of both concrete and abstract words, but the temporal patterns differed. For concrete words, music–word valence-congruency effects were mainly observed following negative music primes, with negative words eliciting larger P2 and late positive component amplitudes than positive words. Musical context also modulated the N400 amplitudes, suggesting effects across multiple processing stages. For abstract words, no reliable interaction was observed in the P2 or N400 time windows. Instead, a late-stage valence effect emerged mainly following positive music primes, with negative words eliciting larger late positive component amplitudes than positive words. **Conclusions:** These findings suggest that musical emotion interacts with concrete and abstract word processing through distinct temporal patterns. The results offer preliminary support for the contextual compensation hypothesis, suggesting that concrete words may be more sensitive to external musical contexts during emotional processing, whereas abstract words appear to rely more on later-stage emotional integration. These findings therefore highlight context-dependent effects of musical emotion and music–word valence congruency on concrete and abstract word processing.

## 1. Introduction

Music and language are two central forms of human emotional expression and social communication. Despite marked differences in their physical properties and representational formats, both modalities reliably activate neural networks associated with emotion recognition and evaluation, including the amygdala, anterior cingulate cortex, and orbitofrontal cortex [1,2,3]. This partial overlap in neural substrates provides a basis for cross-modal interactions between music and language. The OPERA hypothesis (Overlap, Precision, Emotion, Repetition, and Attention) further proposes that musical training or musical experience may influence language processing partly because music and language share certain neural resources. In particular, music-induced emotional states may enhance neural excitability and thereby modulate the plasticity of language-processing networks [4]. Consistent with this view, empirical studies have shown that musical contexts can significantly affect subsequent language processing. This effect is especially evident in emotional processing, where reliable cross-modal interactions between music and language have been observed [5,6,7].

Previous research on the influence of music on language processing has mainly focused on the emotional congruency effect, also referred to as the cross-modal emotional priming effect. Specifically, when a preceding musical excerpt and a subsequently presented word are matched in emotional valence, individuals typically exhibit higher accuracy in lexical judgments, shorter reaction times, and reduced N400 amplitudes in ERP measures [7,8,9]. In other words, when the valence of a target word matches the emotion elicited by the musical prime, the word’s affective-semantic representation is assumed to remain relatively highly activated, facilitating both semantic access and integration [10,11].

However, although the cross-modal emotional priming effect of music on language processing has been widely demonstrated, its underlying mechanism remains insufficiently understood. One important issue is that previous studies have often treated words as relatively homogeneous emotional target stimuli. They have primarily examined whether the emotional valence of music and words is congruent, while paying less attention to differences in the representational bases of lexical concepts themselves. In fact, words are not homogeneous linguistic materials. Depending on the experiential basis of conceptual representation, words with different levels of concreteness may differ substantially in the sources of their semantic representations, the relative contribution of emotional experience, and the extent to which emotional networks are engaged [12,13]. Therefore, heterogeneity within lexical concepts may modulate the strength and efficiency with which musical emotion spreads to word representations. In other words, even when music and target words are emotionally congruent, different types of words may not benefit equally from a musical emotional context.

The semantic representation theory proposed by Vigliocco et al. (2009) provides an important theoretical framework for addressing this issue [12]. According to this theory, the semantic construction of concrete and abstract words relies on different sources of experience. The meanings of concrete words are primarily grounded in sensorimotor experiences acquired via interactions with the external world, and their semantic representations depend more heavily on perceptual and action-related information. By contrast, abstract words lack stable and directly perceptible external referents. Their meanings rely more strongly on linguistic experience, social interaction, and internal emotional and interoceptive experiences, with emotional information playing a more central role in their semantic representations [12,13]. Neurobiological evidence is consistent with this account. Previous studies have demonstrated that concrete-word processing is more strongly associated with frontoparietal networks related to sensorimotor processing, whereas abstract-word processing is more likely to engage brain regions involved in emotional processing, such as the rostral anterior cingulate cortex [13,14].

Based on this theory, musical emotional contexts may exert distinct effects on concrete and abstract words. Music is a highly effective medium for emotional expression, as it can rapidly induce emotional states through acoustic cues such as mode, tempo, timbre, and rhythm [2,15,16]. This theoretical framework leads to two plausible but competing predictions. The first prediction is based on the pre-activation account of emotional priming. If musical emotional priming facilitates word processing by pre-activating valence-related conceptual representations, then abstract words may benefit more from such pre-activation when the emotion induced by music is congruent with the emotional valence of the target word. This is because abstract-word processing is thought to rely more strongly on affective and interoceptive information than concrete-word processing. Accordingly, compared with concrete words, abstract words may be more sensitive to emotional contextual modulation and may therefore show a stronger priming effect under music–word emotional congruency. This prediction is consistent with findings from emotional priming studies within the language modality, which have shown that abstract words are more susceptible than concrete words to the influence of emotional context [11,17,18]. If this prediction is supported in a musical priming context, it would suggest that the facilitative role of emotional information in abstract-word processing is not limited to intrinsic lexical features or linguistic contexts but can also extend to cross-modal musical emotional contexts.

However, an alternative prediction can be derived from the perspective of contextual compensation. According to this view, when the information carried by a target stimulus is relatively weak, ambiguous, or insufficient, individuals rely more strongly on external contextual information to support identification and processing [19,20,21]. A comparable compensatory role of contextual information has also been proposed in research on speech-emotion recognition [22]. Applied to the present study, this perspective suggests that concrete words may benefit more from musical emotional contexts. Because concrete-word meanings are primarily grounded in sensorimotor experience, their emotional attributes may be less strongly activated when they are presented in isolation or without a supportive emotional context. In this case, music, as a highly affective nonverbal context, may provide additional emotional input and compensate for the relatively weaker intrinsic emotional grounding of concrete words. If this mechanism holds, concrete words may show greater processing benefits from musical emotional contexts than abstract words. That is, the music–word emotional congruency effect may be more pronounced for concrete words.

To address these issues, we conducted two experiments to investigate how musical emotion influences the processing of concrete words (Experiment 1) and abstract words (Experiment 2). Drawing on the shared representational distinction between concrete and abstract concepts, the study tested two competing hypotheses: the affective pre-activation hypothesis, which predicts stronger emotional-context effects for abstract words, and the contextual compensation hypothesis, which predicts stronger effects for concrete words. Event-related potentials (ERPs), which provide high temporal resolution, were used to reveal the dynamic neural processes through which musical emotional contexts affect word processing across different levels of concreteness. To ensure adequate processing of the musical emotional context, we adopted a long stimulus-onset asynchrony (SOA) priming paradigm, in which auditory musical excerpts were presented before visual lexical stimuli. Our ERP analyses were not restricted to the N400 component, which has been widely reported in previous music–language emotional priming studies [5,6,9,16]. Instead, we examined multiple stages of lexical processing: early attentional and affective evaluation indexed by the P2, semantic access and integration indexed by the N400, and later evaluative and integrative processing indexed by the late positive component (LPC). By examining this full temporal course, the present study aimed to clarify how musical emotional contexts modulate the processing of words with different levels of concreteness and how conceptual representation shapes cross-modal emotional interactions. This design was intended to provide new empirical evidence and theoretical insights into both the supramodal and modality-specific mechanisms of human emotional processing.

## 2. Experiment 1: Musical Emotional Priming of Concrete Words

### 2.1. Materials and Methods

#### 2.1.1. Participants

Sample size was determined a priori through a power analysis performed using G*Power version 3.1.9.7 [23]. Following Cohen’s conventional benchmark for a medium effect size in ANOVA designs [24], and consistent with a recent ERP priming study on Mandarin language processing [25], the effect size was set at f = 0.25. With α = 0.05 and power (1 − β) = 0.80, the analysis indicated that a minimum of 24 participants was required. A total of 26 female university students were initially recruited. Two participants were excluded because of excessively low behavioral accuracy and/or substantial EEG artifacts. The final sample consisted of 24 female university students, aged 18 to 30 years (M = 22.6, SD = 2.50), who received monetary compensation for their participation. All participants were right-handed, native Mandarin speakers with normal hearing. They reported no history of formal musical training and no history of neurological or psychiatric disorders.

Participants’ baseline affective states were assessed using the Positive and Negative Affect Schedule (PANAS) [26]. Mean scores were 30.8 ± 5.4 for Positive Affect (PA) and 22.6 ± 6.4 for Negative Affect (NA). Each subscale ranged from 10 to 50. These scores suggested that participants did not show marked positive or negative affective states before the experiment, indicating a relatively stable baseline emotional state.

#### 2.1.2. Stimuli

##### Word Stimuli

The word stimuli used in the present study were nouns selected from the *Contemporary Chinese Dictionary (7th edition)*. To ensure the validity and appropriateness of the materials, 30 university students were invited to rate the initially selected words on multiple dimensions. Ratings were collected using 9-point Likert scales and covered four dimensions: concreteness, valence, arousal, and familiarity. For the valence ratings, lower scores indicated more negative valence, higher scores indicated more positive valence, and the midpoint of the scale indicated neutral valence. Following Yao et al. (2016) [27], words with concreteness ratings greater than 7 were classified as concrete words. Word frequency values were retrieved from the SUBTLEX-CH corpus established by Cai and Brysbaert (2010) [28]. This corpus is based on 6243 film subtitles. In the present study, word frequency was indexed by the log10-transformed raw frequency. Context availability, defined as the richness of contextual information elicited by the referent of a concept [29], was also obtained from this corpus.

In addition, a forced-choice task was conducted to determine the basic emotional category of each word. Raters were asked to classify each word into one of four categories: “happy,” “threatening,” “sad,” or “none of the above.” The emotional category selected by more than two-thirds of the raters was taken as the basic emotion of that word. Specifically, words for which more than two-thirds of the raters selected “happy” were classified as positive words, whereas words for which more than two-thirds of the raters selected either “threatening” or “sad” were categorized as negative words.

The use of one positive category and two negative categories was guided by the density hypothesis and related valence-density accounts [30,31], which propose that positive information tends to be more similar and densely interconnected, whereas negative information is more heterogeneous and distributed across more diverse semantic categories. Accordingly, a single positive category such as happiness may capture a relatively coherent positive-valence space, whereas negative valence may require more than one category to represent its broader semantic distribution. Therefore, both sadness-related and threat-related words were included as subcategories of negative valence.

After screening, 24 positive concrete words and 24 negative concrete words were retained. The negative word set included 7 sadness-related words and 17 threat-related words. The imbalance between the numbers of sadness-related and threat-related words was mainly due to the limited availability of sadness-related concrete nouns that reliably conveyed sadness and simultaneously met the selection criteria for concreteness, valence, arousal, familiarity, word frequency, context availability, and character stroke count. Because the present study focused on positive versus negative valence rather than on differences between specific negative emotion categories, sadness-related and threat-related words were combined into a single negative-valence condition in the main analyses.

Independent samples *t*-tests revealed that valence ratings were significantly higher for positive words than for negative words. The two word groups were well matched in concreteness, arousal, familiarity, word frequency, context availability, and character stroke count, with no significant differences observed for these variables. Descriptive statistics (means ± standard deviations) and *t*-test results are summarized in Table 1.

##### Music Stimuli

The musical stimuli were selected from a set of pure piano instrumental excerpts composed and normatively rated for emotional valence by Vieillard et al. (2008) [32]. Notably, this musical corpus has been successfully employed in previous studies investigating cross-modal emotional priming between music and words [7]. Following the same valence-category structure used for the word stimuli, 24 positive-valence musical stimuli, referred to as “happy excerpts,” and 24 negative-valence musical stimuli were selected. The negative musical stimulus set included 7 sad excerpts and 17 threatening excerpts. This distribution was adopted to maintain consistency with the word-stimulus categories while ensuring a sufficient number of negative-valence trials. Each musical excerpt lasted 3 s. The music materials were digitized at 16-bit resolution and a sampling rate of 22.05 kHz, with root mean square (RMS) amplitudes normalized to 70 dB sound pressure level (SPL).

##### Stimulus Pairing

The experiment adopted a 2 (music valence: positive, negative) × 2 (word valence: positive, negative) within-subject design. According to the principle of emotional congruency, musical and lexical stimuli were paired to form four experimental conditions: positive music–positive word (PP), positive music–negative word (PN), negative music–positive word (NP), and negative music–negative word (NN). In these abbreviations, the first letter denotes music valence and the second letter denotes word valence.

Each condition included 24 trials, resulting in a total of 96 trials, and all participants completed trials from all four conditions. Specifically, the PP condition consisted of 24 pairings of happy musical excerpts and happy words. The NN condition included 7 pairings of sad music with sad words and 17 pairings of threatening music with threatening words. The PN condition consisted of 24 pairings of happy music with negative words, including 7 sadness-related words and 17 threat-related words. The NP condition consisted of 24 pairings of negative music with happy words, including 7 sad excerpts and 17 threatening excerpts. Trials from the four conditions were intermixed and presented in a randomized order for each participant, with the constraint that no more than two consecutive trials from the same condition could occur.

#### 2.1.3. Procedure

##### Lexical Valence Judgment Task

The experiment was programmed and presented using E-Prime 3.0 software. Before the experiment, participants received detailed task instructions. After confirming that they had understood the task, they completed a practice session before the formal experiment. Participants were required to achieve an accuracy rate of at least 70% in the practice session before proceeding to the formal experiment. The practice trials followed the same procedure as the formal trials. Each trial proceeded as follows. First, a red fixation cross (“+”) was presented at the center of the screen while a 3000 ms musical excerpt was played. After the music ended, a blank interval of 200 ms was presented, followed by the target word, such as “candy,” for 500 ms. After the word disappeared, a 500 ms blank interval was presented. Participants then entered a 3000 ms response window, during which they were required to judge the emotional valence of the word as quickly as possible, with performance measured by accuracy and response time. They pressed the “4” key if the word was judged as positive, and the “6” key if the word was judged as negative. After the response window, a jittered interval with a mean duration of 1500 ms was presented to reduce temporal predictability across trials.

##### Post-Experimental Strategy Assessment

After the experiment, participants completed a post-experimental strategy assessment adapted from Malhi and Buchanan (2020) [33]. The questionnaire was designed to examine participants’ preferred strategies when judging the valence of concrete words. Specifically, participants rated how frequently they used three types of strategies on a 5-point scale. The first was a visualization/imagery strategy, referring to the use of mental images, scenes, or objects corresponding to the word to make a valence judgment. The second was an emotional/intuitive strategy, referring to judgments based on internal emotional feelings or intuitive experiences elicited by the word. The third was a definition/semantic knowledge strategy, referring to judgments based on the objective definition of the word or commonly held semantic knowledge. Higher scores indicated more frequent use of the corresponding strategy.

#### 2.1.4. EEG Recording and Analysis

EEG signals were recorded using a Neuroscan EEG recording and analysis system. Scalp EEG activity was recorded from a 64-channel wet Ag/AgCl electrode cap based on the extended international 10–20 system. During online recording, the tip of the nose served as the reference electrode. Conductive gel was applied to each electrode to reduce contact impedance, and the scalp at each electrode site was gently prepared and cleaned before gel application. Electrode impedances were kept below 5 kΩ. The total electrode preparation time for each participant was approximately 20 min. EEG signals were sampled at 1000 Hz, with an online band-pass filter of 0.05–100 Hz. Additionally, horizontal electrooculogram (HEOG) and vertical electrooculogram (VEOG) activities were recorded to monitor eye movement artifacts. HEOG electrodes were placed at the outer canthi of both eyes, while VEOG electrodes were positioned above and below the left eye. During offline analysis, the data were re-referenced to the mathematical average of the bilateral mastoids.

Offline EEG preprocessing was conducted using MATLAB R2020a and EEGLAB 2023.0. The continuous EEG data were downsampled and band-pass filtered at 0.1–40 Hz. Bad segments were first removed by visual inspection. Bad channels were identified for each participant during preprocessing based on excessive noise, flat signals, or abnormal activity. When bad channels were present, they were reconstructed using spherical interpolation implemented in EEGLAB, based on the surrounding valid channels. In Experiment 1, an average of 2.5 ± 2.6 channels per participant were interpolated, corresponding to approximately 3.9% ± 4.1% of the 64 recorded channels. Ocular artifacts were corrected using independent component analysis (ICA). ICA components reflecting eye blinks or horizontal eye movements were identified based on their scalp topographies, time courses, and activation patterns. The ICLabel plugin was used as an additional reference for component classification, and the final identification of ocular components was confirmed by visual inspection in EEGLAB. These ocular components were removed, and the remaining components were back-projected to reconstruct the EEG data. EEG epochs were extracted from −200 to 800 ms relative to visual word onset, with word onset defined as time zero. Baseline correction was applied using the 200 ms pre-stimulus interval. After ocular artifact correction, epochs containing residual artifacts, including residual eye movements, large muscle artifacts, abrupt voltage shifts, or other abnormal fluctuations, were excluded from further analyses. In addition, trials with amplitudes exceeding ±100 μV were rejected. Only trials with correct behavioral responses were included in the ERP analyses. For each participant and each condition, ERP waveforms were averaged across correct and artifact-free trials. The mean numbers of accepted trials were reported for each condition in ERP results. To ensure the stability of the ERP waveforms, only conditions in which at least 85% of the correct trials remained after artifact rejection was included in the ERP averaging.

Based on the grand-average waveforms and previous findings [34,35,36,37,38], the following ERP components and time windows were selected for analysis: P2, 200–240 ms; N400, 280–380 ms; and LPC, 385–650 ms. The 200–240 ms interval selected for the P2 was consistent with previous research on affective visual-word processing [39] and encompassed the mean P2 peak latency for every condition of Experiment 1. We adopted a relatively early and narrow time window for the N400 compared with those used in prior studies [5,6,8,9,16], consistent with the early frontocentral negativity evident in our ERP waveforms. This early window also minimized temporal overlap with the subsequent LPC, which began at approximately 385 ms. The electrodes selected for analysis were as follows. For the P2 component, F7, F3, Fz, F4, F8, C3, C1, Cz, C2, and C4 were included. For the N400 component, F3, F1, Fz, F2, F4, FC3, FC1, FCz, FC2, and FC4 were included. For the LPC component, CP3, CP1, CPz, CP2, CP4, P3, P1, Pz, P2, and P4 were included.

For each participant and experimental condition, the mean amplitude of each ERP component was calculated by averaging ERP activity across all time points within the predefined analysis window and across the selected electrodes within the corresponding region of interest. Separate 2 (music valence: positive, negative) × 2 (word valence: positive, negative) repeated-measures ANOVAs were conducted for the P2, N400, and LPC components. Statistical analyses were performed using SPSS 22.0. The Greenhouse–Geisser correction was applied where appropriate, and Bonferroni correction was used for post hoc multiple comparisons. To control the false discovery rate associated with multiple tests across the three ERP components (P2, N400, and LPC), we applied the Benjamini–Hochberg false discovery rate (FDR) correction to the *p* values for the primary interaction effects (music valence × word valence). FDR-adjusted *p* values < 0.05 were considered statistically significant.

### 2.2. Results

#### 2.2.1. Behavioral Results

##### Accuracy and Reaction Time

As described in the Section 2.1.3. Procedure, behavioral performance was assessed using accuracy and response time in the lexical valence judgment task (see Table 2). Trials with no responses and trials with reaction times exceeding 3 standard deviations from the mean were excluded prior to analysis. Accuracy and reaction times were analyzed using separate 2 (music valence: positive, negative) × 2 (word valence: positive, negative) repeated-measures ANOVAs. For accuracy, the main effect of music valence was not significant, *F*_(1, 23)_ = 4.128, *p* = 0.054, *η_P_*^2^ = 0.152. The main effect of word valence was significant, *F*_(1, 23)_ = 6.488, *p* = 0.018, *η_P_*^2^ = 0.220, indicating higher accuracy for negative words than for positive words. The interaction between music valence and word valence was not significant, *F*_(1, 23)_ = 1.478, *p* = 0.236, *η_P_*^2^ = 0.060. For reaction times, neither the main effect of music valence, *F*_(1, 23)_ = 2.556, *p* = 0.124, *η_P_*^2^ = 0.100, nor the main effect of word valence, *F*_(1, 23)_ = 0.819, *p* = 0.375, *η_P_*^2^ = 0.034, was significant. The interaction between music valence and word valence was also not significant, *F*_(1, 23)_ = 0.770, *p* = 0.389, *η_P_*^2^ = 0.032.

##### Valence Judgment Strategies

Participants primarily adopted a visualization/imagery strategy when judging the valence of concrete words. A repeated-measures ANOVA yielded a significant main effect of strategy, *F*_(2, 46)_ = 9.546, *p* = 0.002, *η_P_*^2^ = 0.293. Post hoc tests showed that the visualization/imagery strategy was used significantly more frequently (4.42 ± 0.57) than the emotional/intuitive strategy (3.17 ± 1.03), *p* < 0.001, and the semantic knowledge strategy (3.54 ± 1.19), *p* = 0.019. No significant difference was observed between the emotional/intuitive and semantic knowledge strategies, *p* = 0.946.

#### 2.2.2. ERP Results

Only trials with correct behavioral responses were included in the ERP analyses. For each participant and each condition, we further required that the proportion of invalid epochs among the correct-response epochs did not exceed 15%, corresponding to a retention rate of at least 85% among correct-response epochs. After ocular artifact correction and rejection of residual artifact-contaminated epochs, the mean numbers of accepted trials were 20.2 ± 3.3 for PP, 21.6 ± 2.7 for PN, 19.5 ± 4.2 for NP, and 21.4 ± 2.3 for NN, with ranges of 13–24, 11–24, 9–24, and 13–24 trials, respectively.

Before examining the ERP components, we conducted an additional baseline analysis to address possible concerns regarding apparent prestimulus activity near −200 ms in the ERP waveforms. Because the ERPs were baseline-corrected using the −200 to 0 ms interval, the mean amplitude over the full baseline period was mathematically centered around zero. Therefore, we examined the early prestimulus baseline window from −200 to −100 ms. Mean amplitudes in this window were extracted from the same regions of interest (ROIs) used for the ERP analyses and submitted to 2 × 2 repeated-measures ANOVAs with Music Valence and Word Valence as within-subject factors. No significant main effects or interactions were observed in Experiment 1, all *Fs* ≤ 3.697, all *ps* ≥ 0.067. These results indicate that there were no systematic between-condition differences in the early prestimulus baseline window.

**Figure 1:**
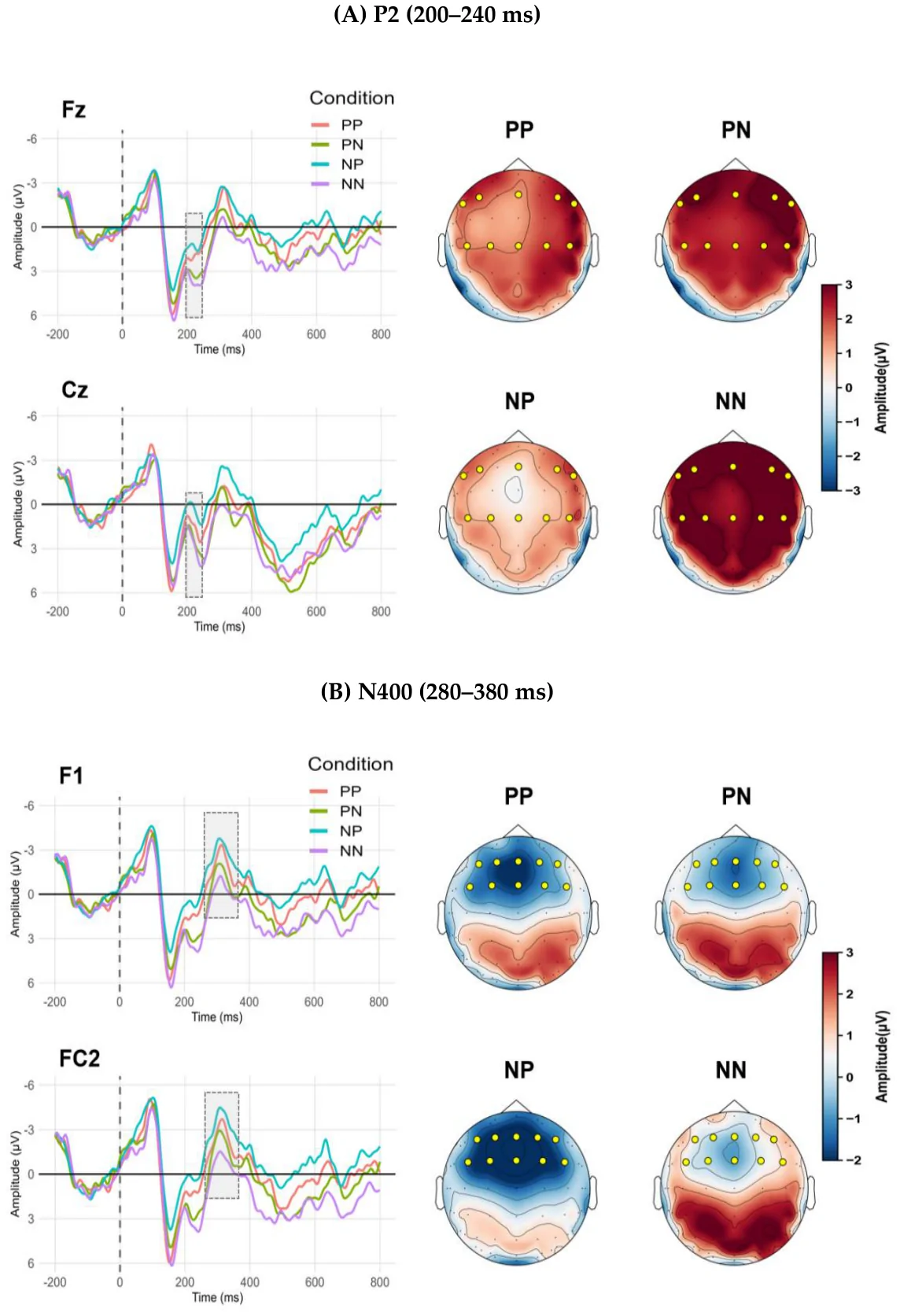

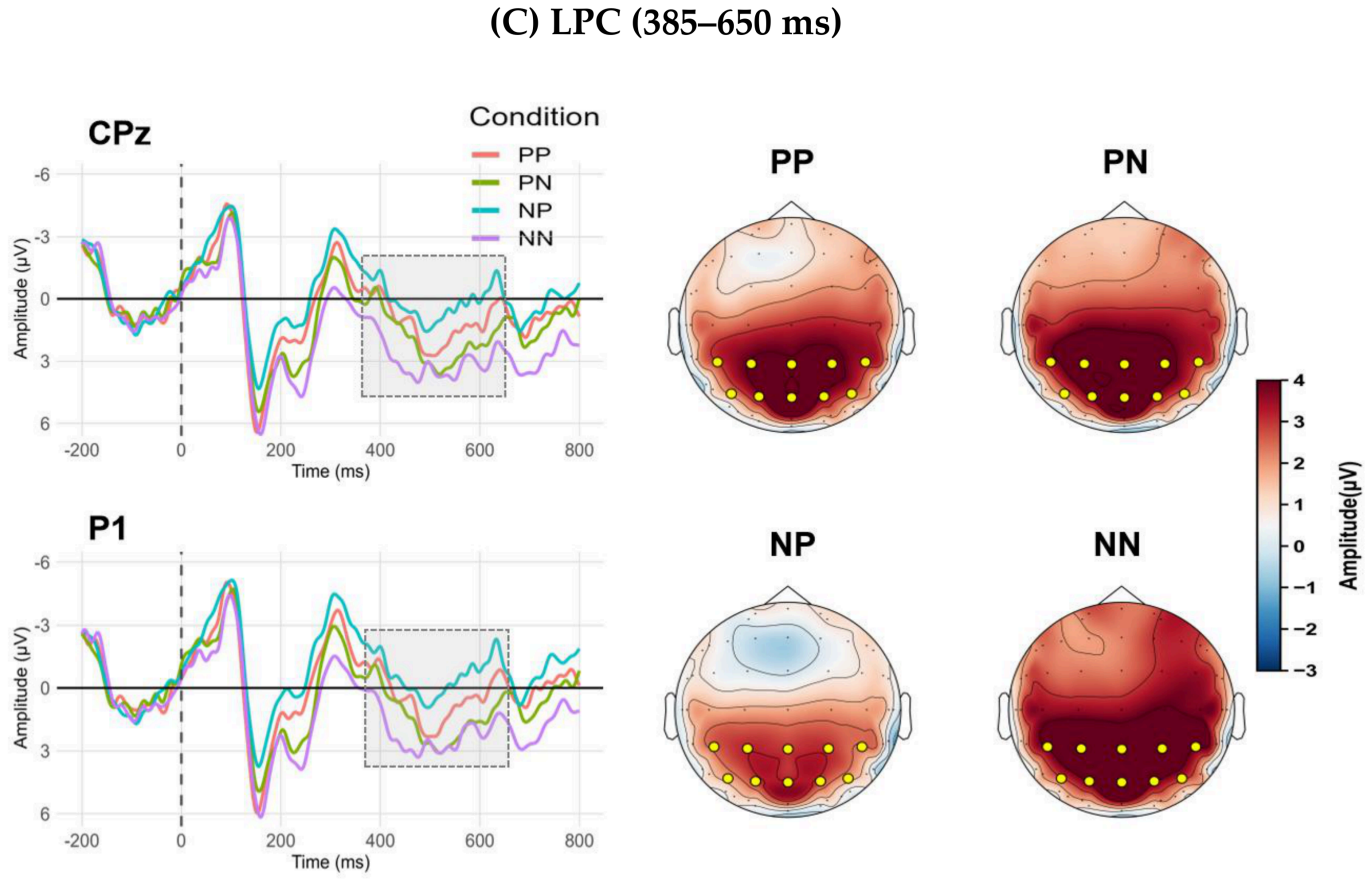
P2, N400, and LPC waveforms and topographic maps in Experiment 1. Panel (**A**) presents ERP waveforms and topographic maps of the P2 component, with Fz and Cz selected as representative electrodes. Panel (**B**) presents ERP waveforms and topographic maps of the N400 component, with F1 and FC2 selected as representative electrodes. Panel (**C**) presents ERP waveforms and topographic maps of the LPC component, with CPz and P1 selected as representative electrodes. The scalp topographic maps were created by averaging the ERP amplitudes over the corresponding analysis time windows shown in the figure: 200–240 ms for P2, 280–380 ms for N400, and 385–650 ms for LPC. Yellow dots on the topographic maps denote electrodes included in the ROIs. Note: PP = positive music–positive word; PN = positive music–negative word; NP = negative music–positive word; NN = negative music–negative word.

We then examined the P2, N400, and LPC components across experimental conditions. Grand-average ERP waveforms and corresponding scalp topographies for the P2, N400, and LPC components are shown in Figure 1.

##### P2

The main effect of music valence was not significant, *F*_(1, 23)_ = 0.095, *p* = 0.761, *η_P_*^2^ = 0.004. The main effect of word valence was significant, *F*_(1, 23)_ = 15.484, *p* = 0.001, *η_P_*^2^ = 0.402, with significantly larger amplitudes observed for negative words relative to positive words. The interaction between music valence and word valence was significant, *F*_(1, 23)_ = 4.940, *p* = 0.036, *p_FDR_* = 0.045, *η_P_*^2^ = 0.177. Post hoc tests showed that the amplitudes did not differ significantly between the PP and PN conditions, *p* = 0.147. However, the NP condition elicited significantly smaller amplitudes than the NN condition, *p* < 0.001.

##### N400

The main effect of music valence was not significant, *F*_(1, 23)_ = 0.057, *p* = 0.813, *η_P_*^2^ = 0.002. The main effect of word valence was significant, *F*_(1, 23)_ = 16.533, *p* < 0.001, *η_P_*^2^ = 0.418, with significantly larger negative-going amplitudes observed for positive words relative to negative words. The interaction between music valence and word valence was also significant, *F*_(1, 23)_ = 5.065, *p* = 0.034, *p_FDR_* = 0.045, *η_P_*^2^ = 0.180. Post hoc tests showed that the amplitudes did not differ significantly between the PP and PN conditions, *p* = 0.236. However, the NP condition elicited significantly larger negative-going amplitudes than the NN condition, *p* < 0.001.

##### LPC

The main effect of music valence was not significant, *F*_(1, 23)_ = 0.933, *p* = 0.344, *η_P_*^2^ = 0.039. The main effect of word valence was significant, *F*_(1, 23)_ = 5.165, *p* = 0.033, *η_P_*^2^ = 0.183, with significantly larger amplitudes observed for negative words relative to positive words. The interaction between music valence and word valence was significant, *F*_(1, 23)_ = 4.498, *p* = 0.045, *p_FDR_* = 0.045, *η_P_*^2^ = 0.164. Post hoc tests showed that the amplitudes did not differ significantly between the PP and PN conditions, *p* = 0.570. However, the NN condition elicited significantly larger amplitudes than the NP condition, *p* = 0.008.

### 2.3. Discussion of Experiment 1

Experiment 1 aimed to examine the effects of emotional musical context and music–word valence congruency on concrete-word processing. Unlike prior studies that mainly reported N400-centered integration effects [9], our ERP results suggested that cross-modal integration may unfold across three successive stages, indexed by the P2, N400, and LPC components.

First, significantly larger P2 amplitudes were elicited by valence-congruent stimuli following negative musical primes within the 200–240 ms time window. This result suggests that musical emotion modulates lexical emotional processing from an early phase. The P2 component has often been associated with stimulus salience and selective attention [40,41]. Accordingly, the P2 interaction observed here suggests that musical emotion may have acted as a salient cue, potentially guiding selective attention toward the emotional attributes of target words. Specifically, when music and words shared the same valence, participants may have shown increased attentional engagement with the emotional features of target words, which could be reflected in changes in P2 amplitudes.

This early P2 effect has not been documented in previous research. One possible explanation for this discrepancy is related to differences in prime duration and stimulus onset asynchrony (SOA). The current study adopted 3000 ms musical excerpts, whereas Goerlich et al. (2012) used shorter 600 ms musical primes [9]. Longer musical excerpts may have allowed participants to build a more stable and well-established emotional context, which could have facilitated cross-modal processing of subsequent lexical emotion. In addition, the present SOA was 3200 ms, much longer than the 200 ms SOA used by Goerlich et al. (2012) [9]. A prolonged SOA may have provided more time for participants to consciously appraise musical emotion and form affective expectations. Such top-down modulation, potentially driven by sustained emotional context, may have promoted the strategic allocation of attention, and thus may have amplified early neural responses to valence-congruent words.

Second, enhanced N400 amplitudes were observed for valence-incongruent trials in the 280–380 ms time window following negative musical primes. This finding aligns with prior emotional priming studies on concrete words, which also reported larger N400 responses to incongruent targets, with the effect predominantly distributed over frontal regions [9]. It may indicate that musical emotion can serve as a robust contextual cue and may generate semantic expectations for subsequent word processing. When target words mismatched the musical context, additional cognitive resources may have been involved in semantic integration, as reflected by elevated N400 amplitudes.

Of note, the N400-related negativity in Experiment 1 was observed within the 280–380 ms time window, with a mean peak latency of 321 ± 29 ms across conditions. Compared with the 400–500 ms N400 window reported by Goerlich et al. (2012) [9], this temporal pattern may suggest that the N400-related activity emerged relatively earlier in the present study. However, because Goerlich et al. did not report N400 peak latencies, this interpretation should be regarded as tentative. We speculate that the potentially shorter N400 latency may be associated with increased attentional engagement in the preceding stage, which could have facilitated the detection of semantic mismatch. As mentioned above, emotional congruency appeared to enhance attention to target words at an early stage. This attentional facilitation likely speeded up subsequent semantic processing and led to earlier detection of semantic incongruity.

Finally, the LPC component (385–650 ms) exhibited enhanced amplitudes for valence-congruent stimuli following negative musical primes. In emotional priming paradigms, the LPC has been linked to late elaborative processing of emotional stimuli, sustained attention and reflective integration [42,43], and it is also sensitive to processing fluency [44]. The present LPC effect may suggest that cross-modal emotional congruency facilitated overall stimulus evaluation and in-depth processing. Long musical excerpts and a prolonged SOA may have allowed participants to form relatively stable emotional expectations. When target words matched the musical valence, cross-modal congruency may have reduced processing difficulty and improved processing fluency. This fluent processing may have been accompanied by additional attentional engagement for deeper semantic encoding and evaluative processing, thereby contributing to enhanced LPC amplitudes. Taken together, these findings may indicate that emotional congruency not only influenced early attentional processing but also supported late-stage integration of emotional meaning.

Despite these ERP modulations, no corresponding effects were observed in behavioral accuracy or reaction time. One possible explanation may be related to the delayed response procedure adopted in the present study. Each target word was presented for 500 ms, followed by a 500 ms blank interval, after which the response prompt appeared and participants made an explicit valence judgment. Thus, the response prompt and motor response occurred after the critical ERP analysis windows, minimizing their potential contamination of the P2, N400, and LPC components. At the same time, because reaction times were measured from the onset of the response prompt, they primarily reflected later decision making and response-execution processes rather than the immediate online processing of the target words. ERP measures may therefore have been more sensitive to subtle and temporally specific effects of music–word emotional congruency that did not persist sufficiently to influence overt behavioral performance.

To summarize, Experiment 1 provided evidence for the impact of musical emotion on concrete word processing. The results suggest that cross-modal integration may be continuously engaged alongside the recognition of lexical valence. The present findings are consistent with the N400 incongruency effect reported in earlier research and further reveal congruency effects on the P2 and LPC components. The P2 and LPC modulations may reflect more efficient processing of words that match the emotional tone of preceding musical contexts. Collectively, these findings suggest that cross-modal emotional integration between music and concrete words may involve a multi-stage process, rather than the single-stage mechanism proposed in some previous studies.

## 3. Experiment 2: Musical Emotional Priming of Abstract Words

### 3.1. Materials and Methods

#### 3.1.1. Participants

Participants in Experiment 2 were recruited using the same inclusion criteria as in Experiment 1. Experiment 2 employed an independent sample distinct from that of Experiment 1. The final sample comprised 24 female university students aged 18–30 years (*M* = 22.4, *SD* = 2.5) who received monetary compensation for their participation. All participants were right-handed native Mandarin speakers with normal hearing, no formal musical training, and no history of neurological or psychiatric disorders. Participants’ baseline affective states were assessed using the PANAS [26]. Mean scores were 29.6 ± 5.9 for PA and 23.5 ± 6.5 for NA, indicating stable emotional states before the experiment.

#### 3.1.2. Stimuli

Consistent with Experiment 1, the word stimuli in Experiment 2 were nouns selected from the *Contemporary Chinese Dictionary (7th edition)*. The initially selected words were rated by 30 university students on multiple dimensions using 9-point Likert scales. The rating dimensions included concreteness, valence, arousal, and familiarity. For the valence ratings, lower scores indicated more negative valence, higher scores indicated more positive valence, and the midpoint of the scale indicated neutral valence. Following Yao et al. (2016) [27], words with concreteness ratings below 4 were classified as abstract words. Word frequency and context availability were retrieved from the SUBTLEX-CH corpus established by Cai and Brysbaert (2010) [28]. The basic emotional category of each word was determined using the same forced-choice procedure as in Experiment 1, in which words were classified as “happy,” “threatening,” “sad,” or “none of the above.”

After screening, 24 positive abstract words and 24 negative abstract words were retained. The positive abstract words were happiness-related words, whereas the negative abstract words included 7 sadness-related words and 17 threat-related words, following the same valence-category structure as in Experiment 1. Independent samples *t*-tests showed that valence ratings were significantly higher for positive words than for negative words. The two word sets were well matched in concreteness, arousal, familiarity, word frequency, context availability, and character stroke count, with no significant differences between groups. Descriptive statistics and *t*-test results are summarized in Table 3.

The musical stimuli used in Experiment 2 were identical to those used in Experiment 1, including 24 happy excerpts as positive-valence musical primes and 24 negative-valence excerpts consisting of 7 sad excerpts and 17 threatening excerpts.

The stimulus pairing procedure was also identical to that in Experiment 1. The experiment adopted a 2 (music valence: positive, negative) × 2 (word valence: positive, negative) within-subject design, forming four conditions: positive music–positive word (PP), positive music–negative word (PN), negative music–positive word (NP), and negative music–negative word (NN). Each condition included 24 trials, resulting in a total of 96 trials. Trials from the four conditions were intermixed and presented in a randomized order for each participant, with the constraint that no more than two consecutive trials from the same condition could occur.

#### 3.1.3. Procedure

The experimental design, lexical valence judgment task, and post-experimental strategy assessment were identical to those used in Experiment 1.

#### 3.1.4. EEG Recording and Analysis

EEG recording and offline preprocessing were identical to those used in Experiment 1. The ERP components, analysis time windows, and electrode regions of interest for the P2, N400, and LPC were also identical to those specified in Experiment 1. In Experiment 2, an average of 2.4 ± 2.1 channels per participant were interpolated, corresponding to approximately 3.8% ± 3.3% of the 64 recorded channels.

### 3.2. Results

#### 3.2.1. Behavioral Results

##### Accuracy and Reaction Time

Trials with no responses and trials with reaction times exceeding 3 standard deviations from the mean were excluded prior to analysis. The mean accuracy and reaction times are presented in Table 4. Accuracy and reaction times were analyzed using separate 2 (music valence: positive, negative) × 2 (word valence: positive, negative) repeated-measures ANOVAs. For accuracy, the main effect of music valence was not significant, *F*_(1, 23)_ = 0.975, *p* = 0.334, *η_P_*^2^ = 0.041. The main effect of word valence was significant, *F*_(1, 23)_ = 4.878, *p* = 0.037, *η_P_*^2^ = 0.175. The interaction between music valence and word valence was not significant, *F*_(1, 23)_ = 3.231, *p* = 0.085, *η_P_*^2^ = 0.123. For reaction times, neither the main effect of music valence, *F*_(1, 23)_ = 1.075, *p* = 0.311, *η_P_*^2^ = 0.045, nor the main effect of word valence, *F*_(1, 23)_ = 0.066, *p* = 0.799, *η_P_*^2^ = 0.003, was significant. The interaction between music valence and word valence was also not significant, *F*_(1, 23)_ = 0.102, *p* = 0.753, *η_P_*^2^ = 0.004.

##### Valence Judgment Strategies

Participants primarily adopted an emotional/intuitive strategy when judging the valence of abstract words. A repeated-measures ANOVA yielded a significant main effect of strategy, *F*_(2, 46)_ = 4.713, *p* = 0.023, *η_P_*^2^ = 0.170. Post hoc tests showed that the emotional/intuitive strategy was used significantly more frequently (3.92 ± 0.86) than the visualization/imagery strategy (3.08 ± 1.22), *p* = 0.008, and the semantic knowledge strategy (3.08 ± 1.04), *p* = 0.024. No significant difference was observed between the visualization/imagery and semantic knowledge strategies, *p* > 0.05.

#### 3.2.2. ERP Results

Only trials with correct behavioral responses were included in the ERP analyses. After ocular artifact correction and rejection of residual artifact-contaminated epochs, the mean numbers of accepted trials were 22.3 ± 2.3 for PP, 23.0 ± 2.1 for PN, 22.1 ± 3.0 for NP, and 23.1 ± 1.8 for NN, with ranges of 15–24, 14–24, 13–24, and 16–24 trials, respectively.

As in Experiment 1, the same baseline analysis was conducted for Experiment 2. Mean amplitudes in the early prestimulus baseline window from −200 to −100 ms were extracted from the same ROIs used for the ERP analyses and submitted to 2 × 2 repeated-measures ANOVAs with Music Valence and Word Valence as within-subject factors. No significant main effects or interactions were observed, all *Fs* ≤ 3.075, all *ps* ≥ 0.093. These results indicated that there were no systematic between-condition differences in the early prestimulus baseline window.We then examined the P2, N400, and LPC components across experimental conditions. Grand-average ERP waveforms and corresponding scalp topographies for the P2, N400, and LPC components are shown in Figure 2. Descriptive statistics for the mean ERP amplitudes and peak latencies across conditions are presented in Table 5 and Table 6, respectively. 

##### P2

The main effect of music valence was not significant, *F*_(1, 23)_ = 2.775, *p* = 0.109, *η_P_*^2^ = 0.108. The main effect of word valence was also not significant, *F*_(1, 23)_ = 2.133, *p* = 0.158, *η_P_*^2^ = 0.085. The interaction between music valence and word valence was not significant, *F*_(1, 23)_ = 0.833, *p* = 0.371, *p_FDR_* = 0.514, *η_P_*^2^ = 0.035.

##### N400

The main effect of music valence was significant, *F*_(1, 23)_ = 8.232, *p* = 0.009, *η_P_*^2^ = 0.264, with significantly larger negative-going amplitudes observed for negative music relative to positive music. The main effect of word valence was not significant, *F*_(1, 23)_ = 0.742, *p* = 0.398, *η_P_*^2^ = 0.031. The interaction between music valence and word valence was not significant, *F*_(1, 23)_ = 0.440, *p* = 0.514, *p_FDR_* = 0.514, *η_P_*^2^ = 0.019.

##### LPC

The main effect of music valence was significant, *F*_(1, 23)_ = 4.875, *p* = 0.037, *η_P_*^2^ = 0.175, with significantly larger amplitudes observed for positive music relative to negative music. The main effect of word valence was not significant, *F*_(1, 23)_ = 0.648, *p* = 0.429, *η_P_*^2^ = 0.027. The interaction between music valence and word valence was significant, *F*_(1, 23)_ = 7.158, *p* = 0.014, *p_FDR_* = 0.042, *η_P_*^2^ = 0.237. Post hoc tests showed that the PN condition elicited significantly larger amplitudes than the PP condition, *p* = 0.047, whereas the NP and NN conditions did not differ significantly, *p* = 0.385. In addition, the PP and NP conditions did not differ significantly, *p* = 0.965, whereas the PN condition elicited significantly larger amplitudes than the NN condition, *p* = 0.001.

### 3.3. Discussion of Experiment 2

Experiment 2 aimed to examine whether musical emotional context modulated the processing of abstract words. Unlike Experiment 1, the most prominent music–word interaction was observed in the LPC time window rather than in the P2 or N400 time windows. Specifically, negative words elicited larger LPC amplitudes following positive musical primes than following negative musical primes, whereas the corresponding difference for positive words was not significant. This pattern suggests that musical emotional context may have modulated later evaluative processing of abstract words. However, this effect was not a general valence-incongruency effect across both music contexts; rather, it was mainly driven by enhanced LPC amplitudes in the PN condition.

The N400 results should be interpreted cautiously. Although a significant main effect of music valence was observed in the N400 time window, neither the main effect of word valence nor the music valence × word valence interaction reached significance. Therefore, the present N400 finding does not provide direct evidence for a music–word emotional congruency effect during semantic processing.

One possible interpretation of the LPC effect is that musical emotional context influenced later stages of affective evaluation or emotional conflict resolution. Previous studies on music–word priming have often reported N400 congruency effects, which have been interpreted as reflecting semantic integration difficulty when target words violate affective or semantic expectations established by music [5,6]. However, those studies differed from the present study in task demands, stimulus materials, and analysis windows. Several earlier studies used semantic relatedness judgment tasks or words closely associated with musical emotion, which may have increased sensitivity to music–word semantic conflict at an earlier stage [8,45]. In contrast, the present task required valence judgments of words that were not directly related to music. Under these conditions, musical primes may have functioned more as a general emotional context than as a source of specific semantic expectations, and the observed modulation may therefore have emerged primarily during later evaluative processing. This interpretation remains tentative.

## 4. Cross-Experiment Combined Analysis

To further examine whether word concreteness moderated cross-modal emotional integration, we combined the data from Experiments 1 and 2 and conducted a mixed-design ANOVA with music valence and word valence as within-subject factors and experiment (concrete vs. abstract words) as a between-subject factor.

The three-way interaction among music valence, word valence, and experiment was significant for the P2 component, *F*_(1, 46)_ = 5.134, *p* = 0.028, *p_FDR_* = 0.042, *η_P_*^2^ = 0.100, and for the N400 component, *F*_(1, 46)_ = 4.309, *p* = 0.044, *p_FDR_* = 0.044, *η_P_*^2^ = 0.086. Follow-up analyses showed that the music valence × word valence interactions in these two components were present for concrete words but not for abstract words. Relevant condition-specific results are reported in Section 2.2.2 and Section 3.2.2.

A significant three-way interaction was also observed for the LPC component, *F*_(1, 46)_ = 11.179, *p* = 0.002, *p_FDR_* = 0.006, *η_P_*^2^ = 0.196. However, the interaction patterns differed between the two word types. For concrete words, negative words elicited larger LPC amplitudes than positive words under negative musical primes, *p* = 0.008, reflecting a congruency-related enhancement. For abstract words, negative words elicited larger LPC amplitudes following positive musical primes than following negative musical primes, *p* = 0.001, reflecting an incongruency-related enhancement. Detailed pairwise comparisons are provided in Section 2.2.2 and Section 3.2.2.

Taken together, these results indicate that word concreteness was associated with different patterns of music–word valence interaction: the P2 and N400 interactions were observed only for concrete words, whereas the LPC interaction was present for both word types but showed different directions.

## 5. General Discussion

The present study explored how musical emotional contexts modulated the processing of concrete and abstract words across two experiments. The ERP results showed that musical emotional contexts modulated the processing of both word types, although the timing and pattern of these effects differed between concrete and abstract words. Prior research has demonstrated that emotional information can be processed implicitly [46,47], and affective priming generally decays rapidly, typically fading around 250 ms after stimulus onset [48]. Consistent with this account, most studies have reported reliable affective priming primarily under short-SOA conditions. Even when priming emerges at long SOAs, its magnitude is usually weaker than that observed with short SOAs [46,47,49]. Notably, these conclusions are largely drawn from within-modality paradigms using words as primes [46,49]. In contrast, the present study detected significant modulation of lexical processing by emotional musical contexts, even under a long-SOA paradigm. This pattern suggests that music may be particularly effective in inducing sustained affective states, which may continue to influence subsequent lexical processing [50].

The most prominent finding is that music appears to influence concrete and abstract words in distinct ways. This result aligns with the hypothesis proposed in the Introduction that lexical conceptual properties may shape cross-modal emotional priming by music. More importantly, the findings offer preliminary support for the contextual compensation hypothesis. Musical influence on abstract words was limited and emerged mainly in the late processing stage. In contrast, music appeared to modulate concrete words across multiple processing stages. These differences are elaborated below.

First, unlike abstract words, concrete words exhibited effects of emotional musical context during the early and middle processing stages: valence-congruent pairs elicited larger P2 amplitudes, while valence-incongruent pairs produced enhanced N400 amplitudes following negative musical primes. These patterns suggest that the effects of emotional musical context on concrete words may involve both early attentional allocation and subsequent semantic analysis. Abstract words, however, showed no reliable modulation by musical context during these stages.

Such divergence may be related to the distinct emotional processing strategies adopted for the two word categories. Questionnaire data suggested that participants mainly relied on a visualization strategy to access the emotion of concrete words. This approach may be relatively time-consuming for lexical valence judgment tasks [33], making concrete words potentially more reliant on contextual compensation for emotional processing. By comparison, post-experimental assessments indicated that participants primarily used an emotional/intuitive strategy to judge the valence of abstract words. This relatively efficient strategy may allow more rapid access to emotional meaning. Furthermore, abstract words are generally considered to possess rich affective connotations and a high emotional load [13,14,51]. Consistent with this view, the current ERP findings did not provide evidence for a significant effect of musical context on early lexical processing.

Second, although musical priming appeared in the late stage for both word types, the direction of the effect differed. For concrete words, larger LPC amplitudes were observed for valence-congruent music–word pairings, with this effect mainly reflected in the NN condition. For abstract words, by contrast, the LPC modulation was mainly reflected in enhanced amplitudes in the PN condition, suggesting that the late effect was not a general valence-incongruency effect across both musical contexts.

One possible interpretation is that the LPC effect for concrete words reflects enhanced late-stage evaluation of emotionally congruent stimuli. Because concrete words had already shown sensitivity to musical context at earlier processing stages, congruent pairings may have received greater attentional engagement and more elaborative evaluation during the later time window. For abstract words, however, the absence of a significant music × word interaction in the P2 and N400 windows suggests that music–word integration may have become evident primarily during later evaluative processing. The enhanced LPC amplitude in the PN condition may therefore reflect increased evaluative demands when negative abstract words were processed after a positive musical context.

This difference in late-stage LPC patterns may reflect distinct ways in which musical emotional context interacted with concrete and abstract word processing. For concrete words, musical context may have provided additional emotional information that supported evaluation across multiple processing stages. For abstract words, the influence of musical context appeared to be more restricted to the later stage, possibly emerging when the affective meaning of the word conflicted with the preceding musical context.

Finally, the present results differ from findings obtained with linguistic emotional contexts. Studies using word primes have shown that lexical emotional priming exerts limited influence on concrete targets but effectively modulates emotional processing of abstract targets, as mainly reflected in LPC responses [18]. The current study revealed a different pattern: musical contexts appeared to produce broader ERP modulations for concrete words than for abstract words.

These two sets of findings are not necessarily contradictory. In within-modality lexical priming, concrete words may provide relatively less affective information than abstract words, which could limit their ability to trigger spreading activation within emotional-semantic networks. As a cross-modal stimulus, however, music may induce affective states through arousal and emotional contagion, thereby providing contextual support for the emotional processing of concrete words. In contrast, because abstract words are often associated with richer affective information, there may be less room for additional modulation by external musical contexts. Accordingly, musical influence on abstract word processing may be relatively constrained.

## 6. Conclusions and Limitations

Previous studies on musical priming have largely overlooked the heterogeneity of lexical conceptual representations. The present study addressed this issue by examining how emotional musical contexts modulate the processing of concrete and abstract emotional words and the associated neural mechanisms. The results showed that musical emotion influenced concrete and abstract words through different temporal courses and neural patterns. These differences may be related to the distinct embodied experiential bases and emotional processing strategies associated with the two types of words. The findings provide a new perspective for understanding the processing mechanisms of emotional language and the role of cross-modal emotional contexts in lexical processing.

Several limitations of this study need to be noted. To begin with, the present study did not include a no-music baseline or a neutral-music control condition. Therefore, the present findings should be interpreted as reflecting the modulation of word processing by the emotional valence of preceding musical contexts, rather than as providing a direct estimate of the absolute effect of musical emotional priming relative to a non-musical or emotionally neutral baseline. Although the observed interactions between musical valence and word valence suggest that emotional congruency influenced ERP responses, future studies should incorporate no-music and/or carefully validated neutral-music conditions to further clarify whether these effects reflect general musical priming, emotional priming, or valence-specific cross-modal congruency.

In addition to the absence of neutral baseline trials, another notable limitation lies in our restricted participant sample. Only female participants were recruited, so the results may not generalize to the wider population. Follow-up research should include male participants and individuals across different age groups to verify the generalizability of the present findings. A further limitation is that the musical stimuli adopted here were not exhaustively controlled for specific acoustic features, such as tempo, pitch, or timbre. Consequently, the current findings may not fully account for how variations in these low-level acoustic properties influence emotional processing. Future research should incorporate a more rigorous analysis and manipulation of these acoustic parameters to disentangle their specific contributions to the emotional interactions between music and language. Finally, the negative-valence condition included both threatening and sad stimuli, which may differ in arousal. Although the same subcategory composition and pairing scheme were used across the two experiments, this within-condition heterogeneity may still have introduced an arousal-related confounder. Future studies should employ more closely matched emotional subcategories or examine threat and sadness separately to better distinguish the effects of valence from those of arousal.

## Figures and Tables

**Figure 2:**
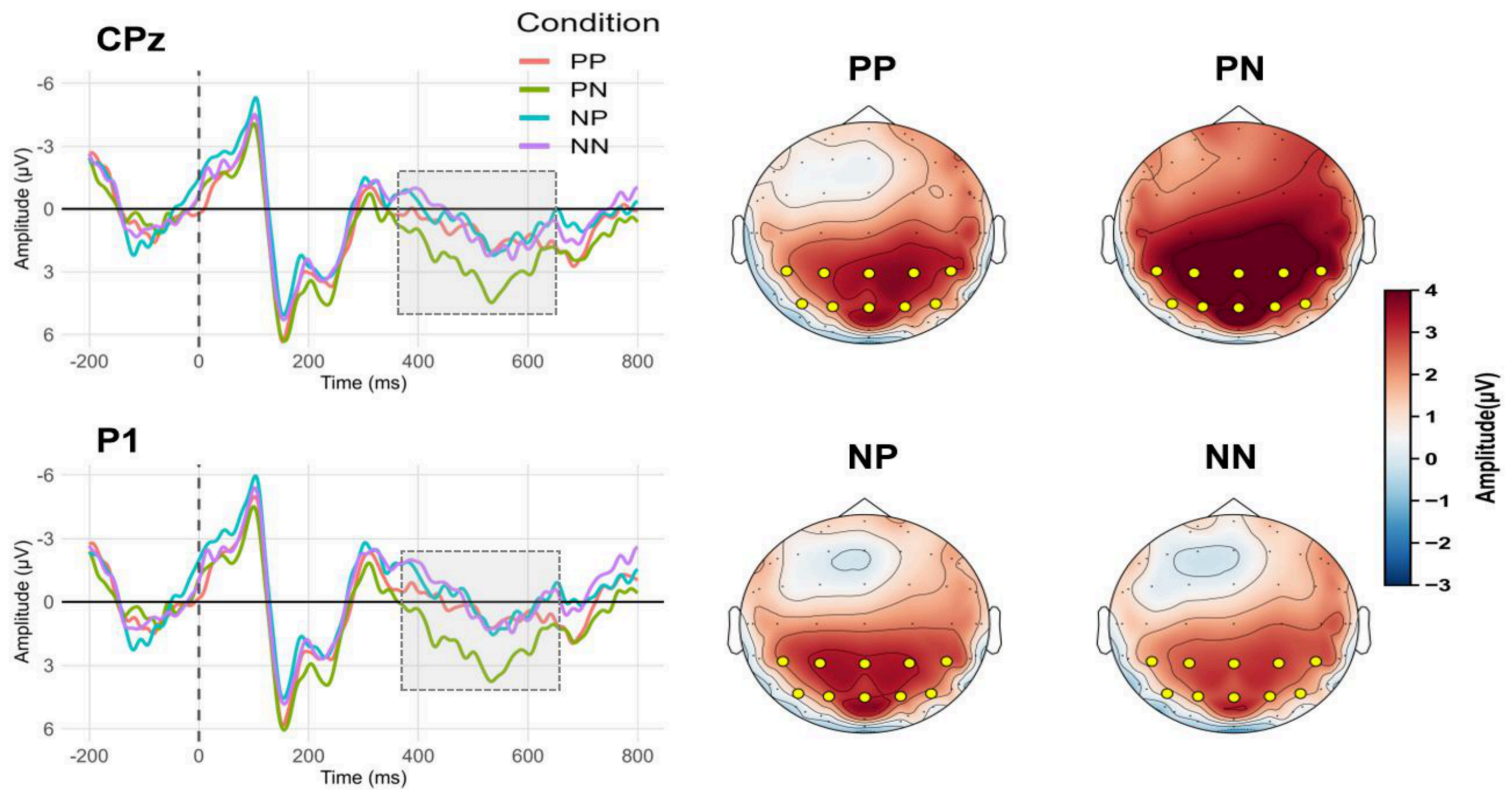
LPC waveforms and scalp topographic maps in Experiment 2. CPz and P1 were selected as representative electrodes. The scalp topographic maps were created by averaging the ERP amplitudes over the corresponding analysis time window shown in the figure: 385–650 ms for the LPC component. Yellow dots on the topographic maps denote electrodes included in the ROIs. Note: PP = positive music–positive word; PN = positive music–negative word; NP = negative music–positive word; NN = negative music–negative word.

**Table 1:** Comparisons between positive and negative concrete words.

Variables	Positive Word	Negative Word	Independent Samples *t*-Tests
Concreteness	8.71 ± 0.37	8.66 ± 0.40	*t*_(46)_ = 0.524, *p* = 0.602, *d* = 0.151
Valence	6.93 ± 0.56	2.70 ± 0.64	*t*_(46)_ = 24.349, *p* < 0.001, *d* = 7.029
Arousal	5.04 ± 1.17	5.54 ± 0.81	*t*_(46)_ = −1.706, *p* = 0.095, *d* = 0.493
Familiarity	8.22 ± 0.38	8.14 ± 0.31	*t*_(46)_ = 0.827, *p* = 0.412, *d* = 0.239
Word frequency	2.78 ± 0.56	2.63 ± 0.39	*t*_(46)_ = 1.026, *p* = 0.310, *d* = 0.296
Context availability	2.45 ± 0.49	2.34 ± 0.34	*t*_(46)_ = 0.882, *p* = 0.382, *d* = 0.255
Character stroke count	18.25 ± 6.13	16.46 ± 5.31	*t*_(46)_ = 1.082, *p* = 0.285, *d* = 0.312

Note. *d* represents Cohen’s *d*.

**Table 2:** Mean accuracy (proportion) and reaction times (ms) for valence judgment of concrete words (*n* = 24).

Conditions	Accuracy	Reaction Times
Positive music–positive word	0.86 ± 0.13	317 ± 94
Positive music–negative word	0.91 ± 0.11	332 ± 107
Negative music–positive word	0.82 ± 0.16	332 ± 98
Negative music–negative word	0.91 ± 0.09	332 ± 101

**Table 3:** Comparisons between positive and negative abstract words.

Variables	Positive Word	Negative Word	Independent Samples *t*-Tests
Concreteness	2.93 ± 0.43	3.06 ± 0.59	*t*_(46)_ = −0.888, *p* = 0.379, *d* = 0.256
Valence	7.05 ± 0.33	2.45 ± 0.47	*t*_(46)_ = 39.024, *p* < 0.001, *d* = 11.265
Arousal	5.17 ± 0.83	5.20 ± 1.07	*t*_(46)_ = −0.116, *p* = 0.909, *d* = 0.033
Familiarity	8.16 ± 0.28	8.20 ± 0.43	*t*_(46)_ = −0.450, *p* = 0.655, *d* = 0.130
Word frequency	2.73 ± 0.51	2.41 ± 0.67	*t*_(46)_ = 1.854, *p* = 0.070, *d* = 0.535
Context availability	2.58 ± 0.43	2.28 ± 0.66	*t*_(46)_ = 1.871, *p* = 0.069, *d* = 0.540
Character stroke count	17.75 ± 5.01	17.83 ± 4.29	*t*_(46)_ = −0.062, *p* = 0.951, *d* = 0.018

Note. *d* represents Cohen’s *d*.

**Table 4:** Mean accuracy (proportion) and reaction times (ms) for valence judgment of abstract words (*n* = 24).

Conditions	Accuracy	Reaction Times
Positive music–positive word	0.95 ± 0.09	316 ± 126
Positive music–negative word	0.97 ± 0.08	311 ± 97
Negative music–positive word	0.93 ± 0.12	323 ± 170
Negative music–negative word	0.97 ± 0.07	322 ± 120

**Table 5:** Mean ERP amplitudes (μV) for each condition across components in Experiments 1 (*n* = 24) and 2 (*n* = 24).

	Component	PP	PN	NP	NN
**Experiment 1**	P2	1.96 ± 4.38	2.76 ± 3.56	1.15 ± 3.56	3.32 ± 3.05
	N400	−1.29 ± 4.92	−0.66 ± 4.58	−2.24 ± 4.32	0.09 ± 4.13
	LPC	3.60 ± 3.75	3.85 ± 3.39	2.71 ± 3.79	4.12 ± 3.32
**Experiment 2**	P2	2.98 ± 3.09	3.62 ± 3.43	2.77 ± 3.51	2.91 ± 3.85
	N400	−0.31 ± 4.78	0.23 ± 4.69	−1.05 ± 4.50	−1.00 ± 4.43
	LPC	2.77 ± 3.86	3.69 ± 3.36	2.75 ± 3.47	2.39 ± 2.47

Note. Condition abbreviations: PP = positive music–positive word; PN = positive music–negative word; NP = negative music–positive word; NN = negative music–negative word.

**Table 6:** Mean peak latency (ms) for each condition across components in Experiments 1 (*n* = 24) and 2 (*n* = 24).

	Component	PP	PN	NP	NN
**Experiment 1**	P2	221 ± 15	224 ± 15	223 ± 15	225 ± 14
	N400	317 ± 27	318 ± 30	323 ± 26	327 ± 31
	LPC	494 ± 46	515 ± 41	493 ± 46	489 ± 53
**Experiment 2**	P2	223 ± 16	227 ± 15	224 ± 15	222 ± 14
	N400	327 ± 32	327 ± 28	325 ± 34	334 ± 36
	LPC	497 ± 63	521 ± 69	512 ± 65	516 ± 59

Note. Condition abbreviations: PP = positive music–positive word; PN = positive music–negative word; NP = negative music–positive word; NN = negative music–negative word.

## Data Availability

The original data presented in the study are openly available on FigShare at http://doi.org/10.6084/m9.figshare.32335188.

## Abbreviations

The following abbreviations are used in this manuscript:

ERP	Event-related potential
FDR	Benjamini–Hochberg False Discovery Rate
kHz	Kilohertz
RMS	Root mean square
ROIs	Regions of interest
SPL	Sound Pressure Level
LPC	Late positive component
PANAS	Positive and negative affect schedule
PA	Positive affect
NA	Negative affect
PP	Positive music–positive word
PN	Positive music–negative word
NP	Negative music–positive word
NN	Negative music–negative word
